# Microscopic Hematuria at Kidney Donor Screening and Post-Donation Kidney Outcomes

**DOI:** 10.3390/jcm11216281

**Published:** 2022-10-25

**Authors:** Jessica van der Weijden, Marco van Londen, Robert A. Pol, Jan-Stephan F. Sanders, Gerjan Navis, Ilja M. Nolte, Martin H. de Borst, Stefan P. Berger

**Affiliations:** 1 Department of Internal Medicine, Division of Nephrology, University Medical Center Groningen and University of Groningen, 9713 GZ Groningen, The Netherlands; 2 Department of Surgery, University Medical Center Groningen and University of Groningen, 9713 GZ Groningen, The Netherlands; 3 Department of Epidemiology, University Medical Center Groningen and University of Groningen, 9713 GZ Groningen, The Netherlands

**Keywords:** microscopic hematuria, living kidney donation, kidney biopsy, living kidney donor evaluation, post-donation outcomes, transplantation

## Abstract

Although guidelines recommend a kidney biopsy in prospective living kidney donors with unexplained microscopic hematuria, individuals with mild hematuria are commonly allowed to donate without a biopsy. However, the prognostic implications of pre-donation hematuria are unclear. We investigated whether pre-donation microscopic hematuria is associated with changes in post-donation eGFR, proteinuria, or blood pressure. We included 701 living kidney donors with two pre-donation urinalyses and post-donation annual evaluations of the estimated glomerular filtration rate (eGFR), protein/creatinine ratio (PCR), and systolic blood pressure (SBP). The association between pre-donation microscopic hematuria and outcomes was assessed using generalized linear mixed models. The median [interquartile range] follow-up was 5 (2–8) years. Eighty-eight donors had pre-donation microscopic hematuria. There were no significant associations between microscopic hematuria at screening and the course of eGFR (0.44 mL/min/1.73 m^2^ increase/year for hematuria donors vs. 0.34 mL/min/1.73 m^2^ increase/year for non-hematuria donors (*p* = 0.65)), PCR (0.02 vs. 0.04 mg/mmol increase/year, *p* = 0.38), or SBP (1.42 vs. 0.92 mmHg increase/year, *p* = 0.17) post-donation, even after adjusting for potential confounders. Additional analyses in high-risk subgroups yielded similar results. In this study, pre-donation microscopic hematuria was not associated with post-donation eGFR decline, proteinuria, or hypertension. Microscopic hematuria may reflect primary kidney disease in only a limited subset of donors. Future studies should identify high-risk donor profiles that require further investigation.

## 1. Introduction

Potential living kidney donors undergo extensive evaluation to minimize the risk of post-donation adverse outcomes. Microscopic hematuria is a common finding during donor evaluation since it affects 8–21% of the general population [1,2]. If a urological evaluation is negative, guidelines advise to exclude glomerular causes by kidney biopsy [3,4]. Common glomerular causes of microscopic hematuria include thin basement membrane nephropathy (TBMN), Alport syndrome or a carrier state, and immunoglobulin A (IgA) nephropathy. While TBMN is the most common cause and generally has an excellent prognosis, Alport syndrome and IgA nephropathy are both associated with an increased risk of developing end-stage kidney disease (ESKD) [5,6,7]. Outside the situation of a potential donation, individuals with microscopic hematuria without additional risk factors suggestive of glomerular disease (i.e., proteinuria, increased serum creatinine levels, or hypertension) generally do not undergo kidney biopsy because the renal prognosis is favorable and the biopsy would have no clinical consequences [8,9]. It is not clear whether the prognosis of microscopic hematuria is also favorable in the setting of living kidney donation. There have been some studies on the effect of hematuria on post-donation outcomes, with variable results, and the studies were mostly on a small scale or had limited follow-up [5,10,11,12,13]. Nevertheless, most of these studies agree on the need of a kidney biopsy to exclude glomerular causes before a potential donor can be accepted for donation. In our center, kidney biopsies are not part of the routine living kidney donor evaluation, and therefore in this study we aimed to evaluate whether microscopic hematuria at donor screening is associated with changes in the post-donation course of proteinuria, eGFR, or blood pressure.

## 2. Materials and Methods

### 2.1. Study Population

In this prospective cohort study, 701 living kidney donors that donated between 1995 and 2018 in the University Medical Center Groningen were included. We included adult donors who provided informed consent and had undergone at least two urinalyses before and/or shortly after donation. Donors with only dipstick measurements and no erythrocyte counts were excluded. None of the donors underwent kidney biopsy. The clinical parameters of weight, height, and blood pressure and laboratory measures, including serum glucose, were measured at baseline. The studies involving human participants were reviewed and approved by the institutional ethical review board. The participants provided written informed consent to participate in this study. All procedures were conducted in accordance with the institutional and national ethical standards and the Declaration of Helsinki, as revised in 2013, and the Declaration of Istanbul.

### 2.2. Urinalyses and Definition of Microscopic Hematuria

Microscopic hematuria was defined as ≥1 red blood cell per high-power field (HPF) or ≥3 red blood cells per µL [11]. Microscopic hematuria was judged as present if it was present at least twice within one year before donation or if it was present at least once within one year before donation and once between three months and one year after donation.

### 2.3. Post-Donation Outcomes

After donation, the urinary protein, estimated glomerular filtration rate (eGFR), and systolic blood pressure (SBP) were measured yearly. We used spot urine from freshly voided urine to measure urinary protein and creatinine and calculated the protein/creatinine ratio (PCR) [14]. Serum creatinine was measured by isotope dilution mass spectrometry that was traceable in our biochemical laboratory by enzymatic assay on the Roche Modular (Roche Ltd., Mannheim, Germany) from 1st March 2006. Before this date, samples were measured by the Jaffe alkaline picrate assay on the Merck Mega Analyzer (Merck, Darmstadt, Germany). Values obtained by the Jaffe method were converted to allow comparison with the Roche method by the formula (YRoche = (XJaffe − 8)/1.07) [15]. The CKD-EPI-creatinine formula was used to calculate the estimated glomerular filtration rate (eGFR) [16]. The 15 min automated office measurement was used to determine blood pressure.

### 2.4. Statistical Analyses

Variables with skewed distributions were naturally log-transformed. Because of repeated measurements, we used generalized linear mixed models to investigate the association between pre-donation microscopic hematuria and the changes in post-donation PCR, eGFR, and SBP over time, using individuals as a random effect and an autoregressive covariance structure. We included the interaction term (hematuria×time) to test whether pre-donation hematuria modified the changes in PCR, eGFR, and SBP over time. The models were adjusted for potential pre-donation confounders, including age, sex, blood pressure, body mass index (BMI), eGFR, PCR, and the use of antihypertensive medication.

For the main analyses, we used ≥ 1 red blood cell per high-power field (HPF) or ≥3 red blood cells per µL as the cut-offs for microscopic hematuria based on a prior study [11], but because other studies used 2–5 red blood cells per high-power field [10,11,12,13,17], we performed sensitivity analyses in which only donors with ≥2 red blood cells per HPF (≥6 per µL) and analyses in which donors with ≥3 per HPF (≥15 per µL) were categorized as “hematuria”. We subsequently defined a subgroup of “high-risk” donors and repeated the generalized linear mixed model analyses in this subgroup. Donors were classified as “high risk” if they had at least one of the following risk factors at screening: SBP > 140 mmHG and/or the use of antihypertensive medication, eGFR < age-adapted threshold [18], PCR > 15 mg/mmol, HbA1c > 7%, or BMI > 30. In further sensitivity analyses, we used uni- and multivariable linear regression analyses to investigate the association between pre-donation hematuria and the five-year post-donation eGFR. Lastly, we used latent class growth modeling in an effort to identify a subgroup of patients with a worse progression of the three outcomes over time. A detailed description of the latent class growth analysis is provided below. For each outcome, we coded the group that showed a worse progression over time as “1” and the group that showed a better progression as “0”. In uni- and multivariable logistic regression analyses, we investigated whether pre-donation microscopic hematuria predicted a worse progression over time for each outcome. SPSS statistics version 22 (IBM, Armonk, NY, USA) and R version 4.0.4 were used to perform the analyses. *p* values < 0.05 were considered statistically significant.

## 3. Results

### 3.1. Pre-Donation Characteristics of the Living Kidney Donor Population

**Figure 1:**
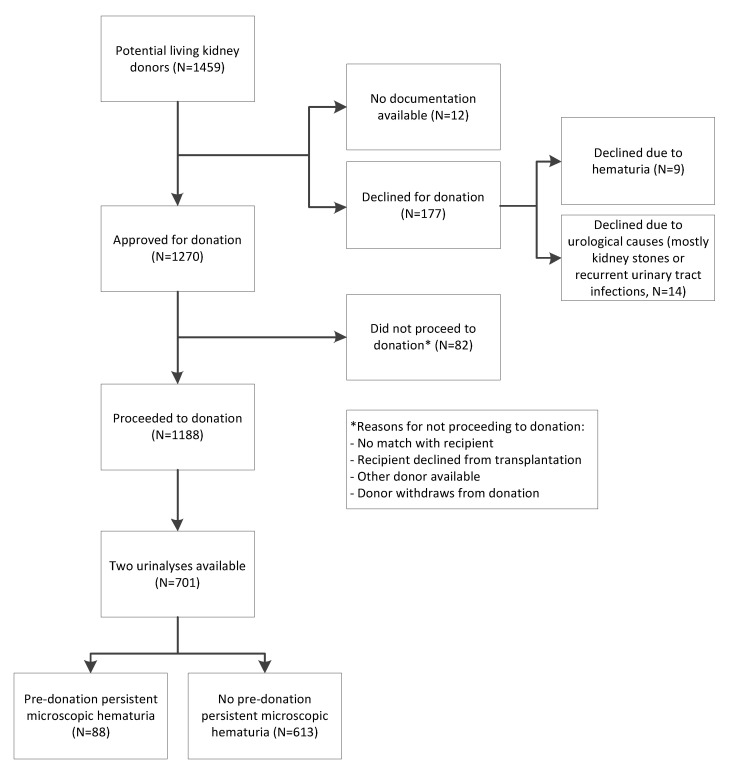
Overview of the study population selection.

A total of 177 donors were excluded from donation, of which 9 donors were excluded due to hematuria (Figure 1). Details of these donors are shown in Table S1. We included 88 (13%) donors with and 613 (87%) donors without hematuria at donor screening (characteristics in Table 1). In donors with hematuria, the median [interquartile range] urinary erythrocyte count was 10 [6,7,8,9,10,11,12,13,14,15,16,17,18,19,20,21,22] per µL. In three donors with hematuria, the medical records documented urological analyses, and in all three cases urological causes were excluded. One donor with hematuria had a known history of nephrolithiasis, but no stones were detected at the time of evaluation. In the hematuria group, 38 (43%) donors were relatives of their recipients. The causes of kidney failure in these recipients are shown in Table S2. The donor age at donation was 54 (11) in the hematuria group and 52 (11) years in the non-hematuria group (*p* = 0.18). The hematuria group consisted of more female donors (*n* = 70 (80%)) than the non-hematuria group (45%, *p* < 0.001). Of these donors, 44 (63%) were >51 years old. Moreover, donors with hematuria had a higher PCR (9 (0–15) mg/mmol) than donors without hematuria (0 (0–12) mg/mmol, *p* = 0.03). The pre-donation eGFR was similar among the two groups (88 (13) mL/min/1.73 m^2^ in the hematuria group vs. 89 (14) mL/min/1.73 m^2^ in the non-hematuria group, *p* = 0.62), as was the SBP (125 (11) mmHg in the hematuria group vs. 127 (13) mmHg in the non-hematuria group, *p* = 0.30.). In the hematuria group, 68 out of the 88 donors had microscopic hematuria twice within one year before donation, and 20 had microscopic hematuria once within one year before donation and once between three months and one year after donation. There were no clinically important significant differences in the characteristics between these two subgroups (Table S3).

### 3.2. Post-Donation Outcomes

**Figure 2:**
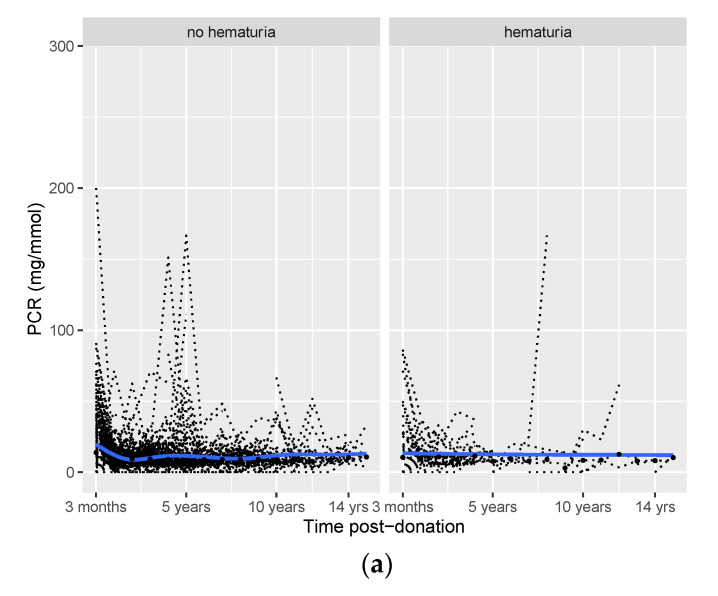

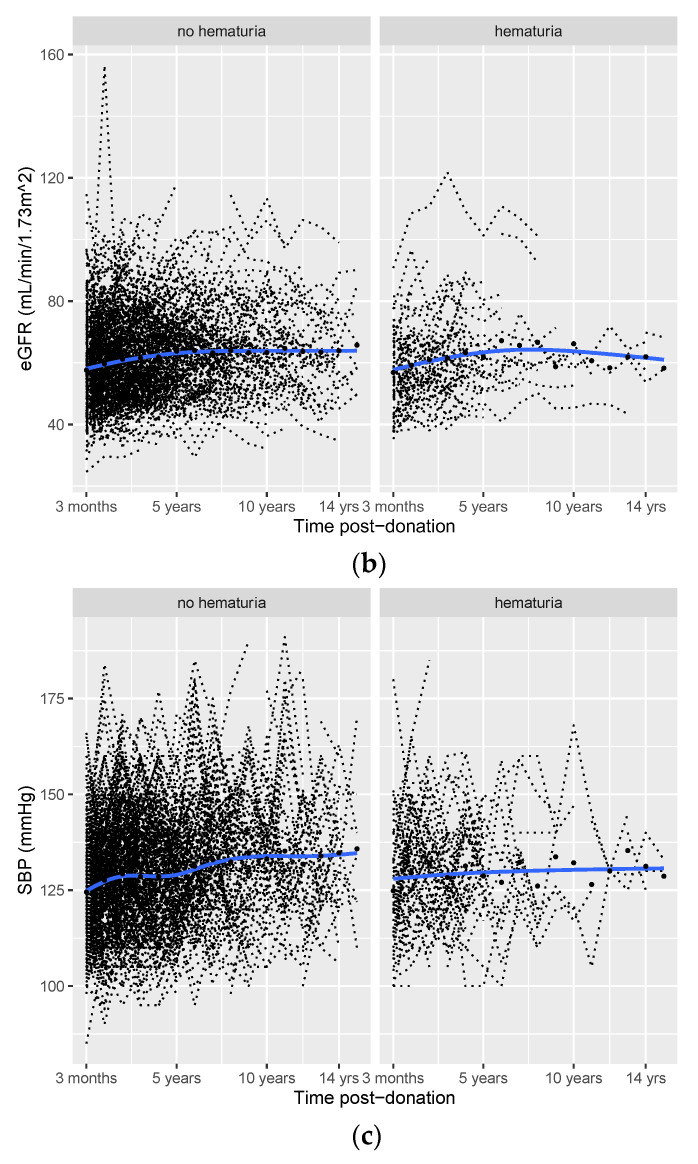
Post-donation course of protein/creatinine ratio (PCR (**a**)), eGFR (**b**), and SBP (**c**) in donors with vs. without pre-donation hematuria. Smooth curves (blue lines) with 95% confidence intervals (grey areas), individual trajectories (dashed black lines), and mean (median for PCR) values for each time point (black dots).

The donor follow-up time was 5 (2–8) years (Figure 2). The last available PCR was moderately increased (15–50 mg/mmol) in 121 donors, of whom 15 (12.4%) had pre-donation microscopic hematuria. In 43 donors, the last measured eGFR was <45 mL/min/1.73 m^2^, of whom 6 (13.9%) had pre-donation microscopic hematuria. For 195 donors, the last measured SBP was ≥140 mmHg, of which 24 (12.4%) donors had pre-donation microscopic hematuria. The prevalence of microscopic hematuria in these groups was not increased compared to the donors without these outcomes, the total population, or the general population [1,2].

### 3.3. Effect of Hematuria on Long-Term Post-Donation Proteinuria, SBP, and eGFR Course

The mean/median values of post-donation PCR, eGFR, and SBP over time are provided in Table S4. The post-donation courses of PCR, eGFR, and SBP were similar among donors with hematuria and those without hematuria (Figure 1). Potential differences between the two groups for the three outcomes over time were tested in generalized linear mixed models (Table 2). In this table, the upper number (hematuria) represents the difference between the hematuria and non-hematuria group at the first visit after donation (at three months). Time represents the course of the outcome after three months for the non-hematuria group, and time*hematuria represents the difference in the post-donation course of the outcome between the hematuria and non-hematuria group. Three months after donation, PCR was 0.28 mg/mmol higher in donors with pre-donation hematuria vs. donors without pre-donation hematuria (*p* = 0.05). However, after three months, PCR increased by 0.04 mg/mmol per year in donors with no pre-donation hematuria (*p* < 0.001), while it only increased by 0.02 mg/mmol per year (time + hematuria×time = 0.04 + (−0.02) = 0.02, Table 2) in donors with pre-donation hematuria.

There was no significant difference in eGFR three months after donation between the hematuria and the non-hematuria groups (estimate = −1.17, *p* = 0.36). Subsequently, post-donation eGFR increased significantly by 0.34 mL/min/1.73 m^2^ per year in donors without pre-donation hematuria (*p* < 0.001, Table 2). While post-donation eGFR increased by 0.44 mL/min/1.73 m^2^ per year in donors with pre-donation hematuria (time + hematuria×time = 0.34 + 0.10 = 0.44, Table 2), the difference in the increase was not significant (*p* = 0.65).

Similarly, there was no significant difference in SBP three months after donation (estimate = 1.18, *p* = 0.45). Post-donation SBP increased significantly by 0.92 mmHg per year after donation in donors without pre-donation hematuria (*p* < 0.001, Table 2). In donors with pre-donation hematuria, post-donation SBP increased by 1.42 mmHg per year (time + hematuria×time = 0.92 + 0.50 = 1.42, Table 2). However, the course of post-donation SBP did not differ significantly between the donors with pre-donation hematuria and those without pre-donation hematuria (*p* = 0.17). The number of donors that used antihypertensive medication at each time point did not materially differ over time (Table S5).

### 3.4. Sensitivity Analyses

We performed sensitivity analyses in which only donors with ≥2 red blood cells per high-power field (≥6 per µL, N = 68) or even ≥3 red blood cells per high-power field (≥15 per µL, N = 46) were classified as “hematuria” (Supplementary Materials). The results of the generalized linear mixed model analyses with these cut-offs did not reveal increased risks of worse post-donation PCR, eGFR, or SBP courses (Tables S6 and S7). Similarly, the results did not change when analyses were performed in a subgroup of donors with microscopic hematuria twice before donation (Table S8).

Generalized linear mixed model analyses were repeated in a subgroup of 306 donors with one or more risk-factors before donation (Supplementary Materials and Table 3). The baseline characteristics of this subgroup are shown in Table S9. Pre-donation hematuria was present in 41 (13.4%) of these high-risk donors. Similar to the total cohort, there was no significant difference in the post-donation course of the outcomes between donors with pre-donation microscopic hematuria and donors without pre-donation hematuria (ln(PCR): difference = −0.02 mg/mmol, *p* = 0.66; eGFR: difference = 0.41 mL/min/1.73 m^2^, *p* = 0.17; SBP: difference = 0.002 mmHg, *p* = 0.99).

We performed further sensitivity analyses (Supplementary Materials) in a subgroup of 332 donors in whom an eGFR at 5 years post-donation was available. Of this group, 34 (10.2%) donors had pre-donation microscopic hematuria, which was not associated with eGFR at five years after donation (Table 4).

Lastly, we defined three subgroups with worse progressions of PCR, eGFR, and SBP over time using a latent class growth analysis (Supplementary Materials, Results, and Figures S2–S4). Pre-donation hematuria was not associated with a worse post-donation course of PCR or eGFR after adjusting for age, sex, and pre-donation PCR/eGFR (Table 5).

## 4. Discussion

The present study aimed to investigate whether living kidney donors with pre-donation hematuria were at increased risk of developing post-donation kidney function impairment compared to donors without hematuria. We found no increased risk of developing (progressive) proteinuria in donors with microscopic hematuria at donor screening over a median follow-up time of five years, nor did we find an increased risk of developing an accelerated loss of kidney function or hypertension. Sensitivity analyses in high-risk subgroups showed similar results. These results do not directly support accepting potential donors with hematuria. However, the results pave the way for further studies to identify which donors with hematuria are at increased risk for glomerular disease and would benefit from a kidney biopsy.

The KDIGO guidelines for living kidney donation state that microscopic hematuria requires further evaluation, which may include urinalysis, cystoscopy, a 24 h urine stone panel, or a kidney biopsy. Only donors with a reversible cause may be accepted for donation, and donors with IgA nephropathy should not donate [3]. The British Guidelines for Living Donor Kidney Transplantation state that donors with glomerular disease, detected on kidney biopsy, should not donate, with the possible exception of TBMN [4]. Although individuals with glomerular disease should not donate, it is unclear in how many patients with microscopic hematuria and no other risk factors for kidney disease on a kidney biopsy will reveal glomerular disease. Outside the setting of living kidney donation, there is an increased long-term risk of ESKD for individuals with microscopic hematuria, but the absolute risk remains very low [17]. The management of these patients is usually not altered by the results of a kidney biopsy, and therefore a kidney biopsy is usually not indicated [8,9]. It is unknown if and/or to what extent unilateral donor nephrectomy changes the risks of microscopic hematuria. In this study, we found no increased renal risk for donors with microscopic hematuria. A kidney biopsy was not performed in the donors with hematuria, which seems to be without consequences in at least the first five years after donation. We would not suggest to never perform a kidney biopsy in potential donors with hematuria. However, we think that these results provide a rationale to discuss and study the position of kidney biopsies in the living kidney donor guidelines.

We observed an initial increase in eGFR over the first five years, followed by a stabilization in the years thereafter, in line with previous studies [19,20]. Our findings may seem to disagree with a previous study by Kido et al. in which pre-donation microscopic hematuria was associated with renal function decline and proteinuria after donation [10]. Differences in the compositions of the cohorts may explain this apparent discrepancy. Kido et al. found that only hematuria with dysmorphic red blood cells was associated with renal function decline and proteinuria. Moreover, in the study by Kido et al., follow-up was only two years, after which renal function was not yet in a steady state, hampering the prediction of long-term risks. In a study by Hassan et al., kidney biopsies were performed in 45 donors with microscopic hematuria [13]. In most donors (*n* = 28), the biopsy results were normal, and in the remaining 17 donors the predominant finding was TBMN (*n* = 13). While the risk of developing ESKD due to TBMN is very low [6], there is no consensus on whether individuals with TBMN can donate [11]. Some studies argue that TBMN is associated with hypertension and proteinuria and that in some cases it could be an expression of the carrier state of Alport syndrome [11]. However, another study showed that living donors with TBMN maintain normal renal function without complications for at least 41 months after donation, and therefore donation with TBMN might be safe [21]. This is different for IgA nephropathy and Alport syndrome, two other relatively common causes of microscopic hematuria [5,7]. The predictors of progression to ESKD for IgA nephropathy are hypertension and proteinuria, but without these conditions the risk of progression of the disease is low [22]. In a study by Nieuwhof et al., biopsy results of 49 patients with microscopic hematuria showed that 12 patients had IgA nephropathy, 13 had TBMN, 4 had miscellaneous diseases, and the remaining 20 biopsies were normal [23]. More importantly, kidney function remained stable over a median follow-up of 11 years. Studies that investigated biopsies of prospective living kidney donors with microscopic hematuria rarely reported Alport syndrome as a finding, probably because Alport syndrome manifests in an earlier stage in life, is commonly accompanied by extrarenal manifestations, and usually affects other family members as well [7].

It is noteworthy that the majority of donors with hematuria in this study were female, and the suggestion could be made that contamination due to menstruation played a role. However, adjustment for sex did not reveal any significant association between hematuria and any of the outcomes after donation. Furthermore, the majority of the female donors had a post-menopausal age. Another notable difference between the hematuria group and the non-hematuria group was a higher PCR in donors with hematuria. While the values of PCR in the hematuria group were only “moderately increased” [14], this could potentially increase the post-donation risks of kidney function impairment. Despite this finding, we found no increased post-donation risks for donors with pre-donation microscopic hematuria. Nevertheless, these data are too limited and the follow-up was too short to draw conclusions about safety for donors with microscopic hematuria combined with moderately increased PCR, and therefore we would not encourage living donation in such cases without further assessment or a kidney biopsy. The same applies to donors with microscopic hematuria and co-existing hypertension or living related donors with a positive family history for kidney diseases. The assessment of risks of kidney failure or premature death were hampered due to the absence of these events. The results of the current study do not support the acceptance of donors with hematuria without biopsy, which we would therefore not encourage. However, the results suggest that a biopsy might only be advantageous for a subset of donors. Of course, future studies using pre-implantation biopsies and with longer follow-up are warranted to confirm our results. Therefore, future studies should profile donors with hematuria at high risk for glomerular disease and investigate possibilities for alternative testing for glomerular diseases that are less invasive such as genetic testing [24]. This could contribute to identifying potential donors with microscopic hematuria that can be accepted for donation without undergoing kidney biopsy.

The strengths of this study include the relatively large sample size and the extensive post-donation kidney function measurements. On the other hand, the average follow-up duration was limited to five years, and few donors had follow-up data beyond 10 years post-donation. Future studies with more complete long-term follow-up should confirm our results. Moreover, we cannot exclude selection bias since more compliant donors may have more complete long-term data. At the same time, some uncomplicated donors might prefer follow-up by the primary health care provider rather than returning to the transplant center [25]. However, the fraction of available long-term follow-up data was similar between the hematuria and non-hematuria groups. Another source of selection bias was the non-selection of donors with pre-donation hematuria who were declined for donation. In our cohort, nine donors were declined because of (sometimes amongst other reasons) hematuria, and since these donors did not donate, we were not able to assess the risk for these donors. In five of these donors, underlying kidney/glomerular disease was suspected (dysmorphic cells/hypertension/proteinuria/kidney lesion on CT), and in the remaining four there were other comorbidities besides hematuria. Third, the hematuria group was relatively small compared to the non-hematuria group, especially in the sensitivity analyses, and may have been underpowered to detect a small additional risk. On the other hand, we did not find a trend towards worse outcomes in the hematuria group. Moreover, the percentages of donors with microscopic hematuria were consistent in the total population and the high-risk subgroups and matched the prevalence found in the general population [1,2]. Another limitation is that we did not have access to kidney biopsies and more detailed analyses of the urine sediment, and urological and/or other follow-up data were only documented in a few donors. This limitation especially applies to living related donors with hematuria, who may be even more at risk of kidney disease. Lastly, the study only consisted of Caucasian donors, limiting the generalizability to other populations.

In conclusion, we found no differences in the five-year post-donation courses of proteinuria, kidney function decline, or hypertension between carefully selected living kidney donors with microscopic hematuria at donor screening (13% of the population) and living kidney donors without hematuria. These results do not support the acceptance of potential donors with hematuria without performing a kidney biopsy. However, the results provide a rationale to identify which donors with hematuria are at risk and could benefit from a kidney biopsy.

## Figures and Tables

**Table 1:** Baseline characteristics of the living kidney donor population.

		Microscopic Hematuria	
	Total (n = 701)	Present (n = 88)	Absent (n = 613)
Female sex, n (%)	345 (49)	70 (80)	275 (45) ^c^
Caucasian race, n (%)	701 (100)	88 (100)	613 (100)
Living related donations, n (%)	328 (47)	38 (43)	290 (47)
Age, years	52 (11)	54 (11)	52 (11)
Weight, kg	81 (14)	77 (13)	81 (14) ^c^
Height, cm	175 (9)	171 (9)	175 (9) ^b^
BMI, kg/m^2^	26 (4)	26 (3)	26 (4)
BSA, m^2^	1.96 (0.20)	1.89 (0.18)	1.96 (0.20) ^b^
SBP, mmHg	127 (13)	125 (11)	127 (13)
DBP, mmHg	76 (9)	75 (9)	76 (9)
Hypertension ^a^, n (%)	183 (26)	23 (26)	160 (26)
Use of antihypertensive medication, n (%)	51 (7)	5 (6)	46 (8)
mGFR, mL/min	115 (22)	111 (22)	115 (22) ^b^
mGFR_BSA_, mL/min/1.73 m^2^	102 (16)	101 (16)	102 (16)
eGFR, ml/min/1.73 m^2^	88 (14)	88 (14)	89 (14)
Serum creatinine, µmol/L	78 (14)	72 (11)	78 (14)^c^
Serum glucose, mmol/L	5.3 (0.6)	5.3 (0.5)	5.3 (0.6)
HbA1C, %	5.5 (0.4)	5.5 (0.3)	5.5 (0.4)
Diabetes, n (%)	6 (1)	1 (1)	5 (1)
Serum cholesterol, mmol/L LDL HDL Triglycerides	5.3 (1.0) 3.5 (0.9) 1.6 (0.5) 1.4 (0.9)	5.3 (1.0) 3.4 (1.1) 1.7 (0.5) 1.2 (0.8)	5.4 (1.0) 3.5 (0.9) 1.5 (0.5) 1.4 (0.9) ^b^
Serum urea, mmol/L	5.4 (1.3)	5.3 (1.2)	5.5 (1.3)
Serum potassium, mmol/L	3.9 (0.3)	3.9 (0.3)	3.9 (0.3)
Serum sodium, mmol/L	141 (3)	141 (3)	141 (3) ^b^
Sodium excretion, mmol/24 h	195 (73)	172 (66)	199 (73) ^b^
PCR, mg/mmol	5 (0–12)	9 (0–15)	0 (0–12)
erythrocytes per µL	n.a.	10 (6–22)	n.a.

^a^: SBP >140 mmHg and/or DBP >90 mmHg. ^b^: *p* < 0.05 vs. “present” group. ^c^: *p* < 0.001 vs. “present” group. Data are presented as means (standard deviations) for normally distributed variables and as medians [first quartile—third quartile] for non-normally distributed variables. Abbreviations: BMI: body mass index; BSA: body surface area; SBP: systolic blood pressure; DBP: diastolic blood pressure; eGFR: estimated glomerular filtration rate; PCR: protein/creatinine ratio.

**Table 2:** Linear mixed model analysis of the associations between pre-donation hematuria and post-donation PCR, eGFR, and SBP over time.

	Outcome PCR			Outcome eGFR			Outcome SBP		
	Estimate	95% CI	P	Estimate	95% CI	P	Estimate	95% CI	P
Hematuria ^a^	0.28	−0.01 to 0.56	0.05	−1.17	−3.66 to 1.32	0.36	1.18	−1.86 to 4.22	0.45
Time	0.04	0.03 to 0.05	<0.001	0.34	0.23 to 0.44	<0.001	0.92	0.75 to 1.09	<0.001
Hematuria×time	−0.02	−0.08 to 0.03	0.38	0.10	−0.34 to 0.54	0.65	0.50	−0.21 to 1.21	0.17

^a^ Donors with pre-donation hematuria were defined as 1, and donors with no pre-donation hematuria were defined as 0. Both models were adjusted for pre-donation age, sex, BMI, eGFR, PCR, SBP, and antihypertensive medication use. N total = 701. N hematuria group = 88. N non-hematuria group = 613. Abbreviations: PCR: protein/creatinine ratio; eGFR: estimated glomerular filtration rate; BMI: body mass index; SBP: systolic blood pressure.

**Table 3:** Linear mixed model analysis for the associations between pre-donation hematuria and post-donation ln(PCR), eGFR, and SBP over time in a subgroup of high-risk donors.

	Outcome PCR			Outcome eGFR			Outcome SBP		
	Estimate	95% CI	P	Estimate	95% CI	P	Estimate	95% CI	P
Hematuria ^a^	0.22	−0.19 to 0.64	0.30	−2.64	−6.14 to 0.87	0.14	1.74	−2.45 to 5.92	0.42
Time	0.05	0.03 to 0.06	<0.001	0.24	0.10 to 0.38	0.001	0.80	0.58 to 1.02	<0.001
Hematuria×time	−0.02	−0.10 to 0.06	0.66	0.41	−0.18 to 0.99	0.17	0.002	−0.89 to 0.90	0.996

^a^: donors with hematuria were defied as 1, and donors with no hematuria were defined as 0. Both models were adjusted for pre-donation age, sex, BMI, eGFR, PCR, SBP, and antihypertensive medication use. N total = 306. N hematuria group = 41. N non-hematuria group = 265. Donors were classified as high-risk if one or more of the following CKD risk factors were present: SBP > 140 mmHG and/or the use of antihypertensive medication, eGFR <age-adapted threshold [18], PCR > 15 mg/mmol, HbA1c > 7%, or BMI > 30. Abbreviations: PCR: protein/creatinine ratio; eGFR: estimated glomerular filtration rate; BMI: body mass index; SBP: systolic blood pressure.

**Table 4:** Uni- and multivariable linear regression analyses of pre-donation hematuria and other characteristics with five-year post-donation eGFR.

	Univariable			Multivariable		
	St. β	95% CI	P	St. β	95% CI	P
Age, years	−0.52	−0.64 to −0.45	<0.001	-	-	-
Sex, 1 = female	−0.10	−0.21 to 0.01	0.07	0.01	−0.12 to 0.13	0.93
BMI, kg/m^2^	−0.01	−0.12 to 0.09	0.80	-	-	-
BSA, m^2^	0.07	−0.04 to 0.16	0.22	0.01	−0.11 to 0.12	0.93
eGFR, mL/min/1.73 m^2^	0.59	0.51 to 0.68	<0.001	0.59	0.50 to 0.38	<0.001
SBP, mmHg	−0.08	−0.19 to 0.02	0.13	−0.03	−0.12 to 0.06	0.50
HbA1c, %	−0.08	−0.19 to 0.04	0.19	−0.09	−0.19 to 0.01	0.07
ln(PCR), mg/mmol	−0.07	−0.27 to 0.13	0.49	-	-	-
Hematuria, 1 = positive	−0.05	−0.17 to 0.07	0.40	−0.06	−0.16 to 0.04	0.23

N total = 332. N hematuria = 34. N non-hematuria = 298. Hematuria and other variables with *p* < 0.2 in univariable analyses were added to the multivariable model. Abbreviations: eGFR: estimated glomerular filtration rate; CI: confidence interval; BMI: body mass index; BSA: body surface area; SBP: systolic blood pressure; PCR: protein/creatinine ratio.

**Table 5:** Uni- and multivariable logistic regression analyses of pre-donation hematuria and worse post-donation outcomes.

	Univariable			Multivariable		
	OR	95% CI	P	OR	95% CI	P
Outcome PCR group						
Hematuria, 1 = positive	0.71	0.35 to 0.43	0.34	0.49	0.16 to 1.51	0.22
Age	1.00	0.98 to 1.02	0.72	0.99	0.96 to 1.02	0.62
Female sex	0.85	0.56 to 1.31	0.46	0.91	0.42 to 1.97	0.81
Pre-donation PCR	1.03	0.53 to 2.00	0.93	1.11	0.53 to 2.34	0.78
Outcome eGFR group						
Hematuria, 1 = positive	1.23	0.50 to 3.03	0.65	1.45	0.56 to 3.72	0.44
Age	0.98	0.95 to 1.01	0.10	0.96	0.93 to 0.99	0.01
Female sex	0.84	0.44 to 1.59	0.58	0.73	0.37 to 1.45	0.37
Pre-donation eGFR	0.98	0.96 to 1.00	0.11	0.97	0.94 to 0.99	0.01
Outcome SBP group						
Hematuria, 1 = positive	0.65	0.41 to 1.04	0.07	0.63	0.37 to 1.05	0.07
Age	1.00	0.98 to 1.01	0.62	1.02	1.00 to 1.04	0.02
Female sex	0.95	0.70 to 1.28	0.72	0.69	0.49 to 0.97	0.03
Pre-donation SBP	0.94	0.92 to 0.95	<0.001	0.93	0.92 to 0.95	<0.001

Outcome classification was based on a latent class growth analysis in which a group was defined that performed worse than the other group after donation. The group with the poorest outcomes was defined as “1” in the logistic regression analysis, and the group with the best outcomes was defined as “0”. PCR: best post-donation course N = 485, poorer course N = 103. eGFR: best post-donation course N = 695, poorer course N = 40. SBP: best post-donation course N = 404, poorer course N = 290. Abbreviations: CI: confidence interval; PCR: protein/creatinine ratio; eGFR: estimated glomerular filtration rate; SBP: systolic blood pressure.

## Data Availability

The data that support the findings of this study are available upon reasonable request from the corresponding author (S.P.B.). The data are not publicly available due to the privacy of the research participants.

## Supplementary Materials

The following supporting information can be downloaded at https://www.mdpi.com/article/10.3390/jcm11216281/s1, Table S1: Description of donors who were declined from donation due to hematuria; Table S2: Causes of kidney failure in recipients who were relatives of donors with pre-donation hematuria.; Table S3: Baseline characteristics of the donors with pre-donation microscopic hematuria; Table S4: Long-term follow-up data outcomes; Table S5: Long-term follow-up data antihypertensive medication use; Table S6: Linear mixed model analysis for the association between pre-donation hematuria (≥2 RBC per high powerfield or ≥6 RBC per µL) and post-donation PCR, eGFR and SBP over time; Table S7: Linear mixed model analysis for the association between pre-donation hematuria (≥3 RBC per high powerfield or ≥15 RBC per µL) and post-donation PCR, eGFR and SBP over time; Table S8: Baseline characteristics of the living kidney donor population according to presence of risk factors; Figure S1: Distribution of pre-donation PCR in the hematuria group and the non-hematuria group; Supplementary Results; Figure S2: Latent class growth model of post-donation PCR course; Figure S3: Latent class growth model of post-donation eGFR course; Figure S4: Latent class growth model of post-donation SBP course.

## References

[1] Mohr D., Kenneth P., Offord M., Owen R., Melton J. (1986). Asymptomatic Microhematuria and Urologic Disease: A Population-Based Study. JAMA.

[2] Cohen R., Brown R. (2003). Microscopic Hematuria. N. Engl. J. Med..

[3] Andrews P.A., Lisa B. (2018). British Transplantation Society/Renal Association UK guidelines for living donor kidney transplantation 2018: Summary of updated guidance. Transplantation.

[4] Lentine K., Kasiske B., Levey A., Adams P., Alberú J., Bakr M., Gallon L., Garvey C.A., Guleria S., Li P.K. (2017). KDIGO Clinical Practice Guideline on the Evaluation and Care of Living Kidney Donors. Transplantation.

[5] Vadivel N., Stankovic A., Rennke H.G., Singh A.K. (2007). Accepting prospective kidney donors with asymptomatic urinary abnormalities: Are we shooting in the dark?. Kidney Int..

[6] Savige J., Rana K., Tonna S., Ruzza M., Dagher H., Wang Y. (2003). Thin Basement Membrane Nephropathy. Kidney Int..

[7] Gross O., Weber M., Fries J.W.U., Müller G.A. (2009). Living donor kidney transplantation from relatives with mild urinary abnormalities in Alport syndrome: Long-term risk, benefit and outcome. Nephrol. Dial. Transplant..

[8] Hall C., Bradley R., Kerr A., Attoti R., Peat D. (2004). Clinical value of renal biopsy in patients with asymptomatic microscopic hematuria with and without low-grade proteinuria. Clin. Nephrol..

[9] McGregor D., Lynn K., Bailey R., Robson R., Gardner J. (1998). Clinical audit of the use of renal biopsy in the management of isolated microscopic hematuria. Clin. Nephrol..

[10] Kido R., Shibagaki Y., Iwadoh K., Nakajima I., Fuchinoue S., Fujita T., Teraoka S. (2010). Persistent glomerular hematuria in living kidney donors confers a risk of progressive kidney disease in donors after heminephrectomy. Am. J. Transplant..

[11] Koushik R., Garvey C., Manivel J.C., Matas A.J., Kasiske B.L. (2005). Persistent, asymptomatic, microscopic hematuria in prospective kidney donors. Transplantation.

[12] Choi S.R., Sun I.O., Hong Y.A., Kim H.G., Park H.S., Chung B.H., Choi B.S., Park C.W., Kim Y.S., Yang C.W. (2012). The role of kidney biopsy to determine donation from prospective kidney donors with asymptomatic urinary abnormalities. Transplant. Proc..

[13] Hassan E.A., Ali T.Z., Abdulbaki A., Ibrahim I.A., Almanae H.M., Aleid H.A. (2017). Histopathologic Findings of Potential Kidney Donors With Asymptomatic Microscopic Hematuria: Impact on Donation. Transplant. Proc..

[14] Fisher H., Hsu C., Vitiinghoff E., Lin F., Bansal N. (2013). Comparison of Associations of Urine Protein-Creatinine Ratio Versus Albumin-Creatinine Ratio With Complications of CKD: A Cross-sectional Analysis. Am. J. Kidney Dis..

[15] Van Londen M., Aarts B.M., Deetman P.E., van der Weijden J., Eisenga M.F., Navis G., Bakker S.J., de Borst M.H., van Londen M., on behalf of the NIGRAM Consortium (2017). Post-transplant hypophosphatemia and the risk of death-censored graft failure and mortality after kidney transplantation. Clin. J. Am. Soc. Nephrol..

[16] Levey A.S., Coresh J., Greene T., Stevens L.A., Zhang Y.L., Hendriksen S., Kusek J.W., van Lente F., Chronic Kidney Disease Epidemiology Collaboration (2014). Annals of Internal Medicine Article Using Standardized Serum Creatinine Values in the Modification of Diet in Renal Disease Study Equation for Estimating Glomerular. Ann. Intern. Med..

[17] Vivante A., Afek A., Frenkel-Nir Y., Tzur D., Farfel A., Golan E., Chaiter Y., Shohat T., Skorecki K., Calderon-Margalit R. (2011). Persistent asymptomatic isolated microscopic hematuria in Israeli adolescents and young adults and risk for end-stage renal disease. JAMA-J. Am. Med. Assoc..

[18] Niertransplantatie L.O., van der Heide J.H. (2008). Nederlandse Richtlijn: Evaluatie Van Potentiële Donoren Voor Levende Donor Niertransplantatie.

[19] Lam N.N., Lloyd A., Lentine K.L., Quinn R.R., Ravani P., Hemmelgarn B.R., Klarenbach S., Garg A.X. (2020). Changes in kidney function follow living donor nephrectomy. Kidney Int..

[20] Matas A.J., Vock D.M., Ibrahim H.N. (2018). GFR ≤ 25 years postdonation in living kidney donors with (vs. without) a first-degree relative with ESRD. Am. J. Transplant..

[21] Choi C., Ahn S., Min S.K., Ha J., Ahn C., Kim Y., Lee H., Min S.I. (2018). Midterm outcome of kidney transplantation from donors with thin basement membrane nephropathy. Transplantation.

[22] D’Amico G. (2000). Natural history of idiopathic IgA nephropathy: Role of clinical and histological prognostic factors. Am. J. Kidney Dis..

[23] Nieuwhof C., Doorenbos C., Grave W., de Heer F., de Leeuw P., Zeppenfeldt E., van Breda Vriesman P.J. (1996). A prospective study of the natural history of idiopathic non-proteinuric hematuria. Kidney Int..

[24] De Haan A., Eijgelsheim M., Vogt L., Knoers N.V.A.M. (2019). Diagnostic Yield of Next-Generation Sequencing in Patients with Chronic Kidney Disease of Unknown Etiology. Front. Genet..

[25] Waterman A.D., Dew M.A., Davis C.L., McCabe M., Wainright J.L., Forland C.L., Bolton L., Cooper M. (2013). Living-donor follow-up attitudes and practices in U.S. kidney and liver donor programs. Transplantation.

