# Supplementary material for: Microscopic Hematuria at Kidney Donor Screening and Post-Donation Kidney Outcomes
Source: J Clin Med. 2022 Oct 25;11(21):6281. doi: 10.3390/jcm11216281 (PMC9655639; doi:10.3390/jcm11216281)
Supplement: Supplementary file 1 [file jcm-11-06281-s001.zip › jcm-1906671-supplementary.pdf]

Table of content Supporting Information

Title	*Page*
Supplementary Methods	S2
Table S1. Description of donors who were declined from donation due to hematuria.	S4
Table S2. Causes of kidney failure in recipients who were relatives of donors with pre-donation hematuria.	S5
Table S3. Baseline characteristics of the donors with pre-donation microscopic hematuria	S6
Table S4. Long-term follow-up data outcomes	S7
Table S5. Long-term follow-up data antihypertensive medication use	S8
Table S6. Linear mixed model analysis for the association between pre-donation hematuria (≥2 RBC per high powerfield or ≥6 RBC per µL) and post-donation PCR, eGFR and SBP over time	S9
Table S7. Linear mixed model analysis for the association between pre-donation hematuria (≥3 RBC per high powerfield or ≥15 RBC per µL) and post-donation PCR, eGFR and SBP over time	S10
Table S8. Baseline characteristics of the living kidney donor population according to presence of risk factors	S11
Figure S1. Distribution of pre-donation PCR in the hematuria group and the non-hematuria group.	S12
Supplementary Results	S13
Figure S2. Latent class growth model of post-donation PCR course.	S14
Figure S3. Latent class growth model of post-donation eGFR course.	S15
Figure S4. Latent class growth model of post-donation SBP course.	S16

Supplementary methods

Latent class growth analysis

SBP, eGFR, and PCR were log-transformed to obtain normally distributed outcome variables. Outliers, defined as values deviating more than four standard deviation from the mean, were removed from the dataset.

We used latent class growth modelling aiming to identify a subgroup of patients with a worse progression over time compared to the rest of them. For this purpose the 'hlme' (heterogeneous linear mixed model) function from the R-package 'lcmm' was used. All models included global and class-specific fixed intercepts and linear and quadratic effects of follow-up time as well as covariates confounders age at donation and gender. The outcome variable were log-transformed eGFR, PCR, and SBP. In addition, four different models were compared: 1) no individual random intercepts, linear or quadratric effects of follow-up time; 2) individual random intercepts, but no linear or quadratric effects of follow-up time; 3) individual random intercepts and linear effects of follow-up time, but no quadratic effects; and 4) individual random intercepts and linear and quadratic effects of follow-up time. Analyses were performed with automated grid searches to run the analyses with ten different starting values to avoid local maxima. For each model the optimal number of classes was determined by the analysis showing the lowest Bayesian Information Criterion (BIC). The best-fitting overall model was regarded the one among the four with the lowest BIC.

**Table S1. Description of donors who were declined from donation due to hematuria.**			
**Case number**	**Findings during donor screening**	**Red blood cell count**	**Further evaluation/conclusions**
Individuals in whom hematuria was the only reason for exclusion (suspected renal disease)			
1	Glomerular (50% dysmorphic red blood cells) microscopic hematuria on three separate measurements without reduced kidney function, proteinuria or hypertension.	8/µL, 8/µL and 22/µL	Exclusion from donation. No biopsy advised, follow-up hematuria at transplant center.
2	Hypertension, proteinuria (2g/24h) and hematuria (291/µL). Unknown whether dysmorphic erythrocytes were present. Possibly renal disease.	455/µL in spot urine and 291/µL in 24h urine	Exclusion from donation. No biopsy advised, follow-up hematuria at transplant center.
3	Glomerular microscopic hematuria (90% dysmorphic red blood cells, no red cell casts).	No count documented	Exclusion from dronation. Follow-up genetic testing revealed a carrier status for Alport syndrome.
4	Glomerular microscopic hematuria (20-40% dysmorphic red blood cells, no cylindric cells). No causes were found at urological evaluation.	24/µL and 153/µL	Exclusion from donation. If continuing the donation procedure is desired, kidney biopsy is needed to exclude glomerular disease.
5	Microscopic hematuria twice during screening without proteinuria, hypertension or reduced kidney function. At urological evaluation, a potentially malignant lesion was seen in the right kidney.	20/µL and 34/µL	Exclusion from donation. Follow-up at urologist for lesion right kidney.
Individuals in whom hematuria contributed to the decision of exclusion amongst other reasons			
6	Low mGFR and erythrocytes in urinesediment. Urine sediment was not repeated during screening due to low mGFR (exclusion from donation anyway).	24/µL	Exclusion from donation. At follow-up by general practitioner, hematuria was no longer present.
7	Possibly SLE and microscopic hematuria on two separate measurements without reduced kidney function, proteinuria or hypertension.	8/µL and 22/µL	Exclusion from donation. Follow-up hematuria at general practitioner was advised.
8	Low mGFR and microscopic hematuria (for which urological evauation had been performed years ago which revealed no urological causes). Besides, a lesion in adrenal glands was found on CT. Lastly unhealthy lifestyle (smoking and alcohol).	4/µL and 5/µL	Exclusion from donation. Follow-up of lesion in adrenal glands was advised.
9	Microscopic hematuria at evaluation (>40% dysmorphic red blood cells). Besides, increased M-protein, alterations on ECG and high blood glucose levels were found.	14/µL and 30/µL	Exclusion from donation. Follow-up of the findings is advised in referral hospital.

Table S2. Causes of kidney failure in recipients who were relatives of donors with pre-donation hematuria.

Cause of kidney failure	*N (%)*
Focal segmental glomerulosclerosis	4 (11%)
Diabetic nephropathy	4 (11%)
IgA nephropathy	4 (11%)
Vesicoureteral reflux nephropathy	4 (11%)
Anatomical abnormalities limiting urine outflow*	4 (11%)
Assumed consequence of hypertension	4 (11%)
Polycystic kidney disease	3 (8%)
Etiology unknown	2 (5%)
Microscopic polyangiitis	1 (3%)
Alport syndrome (mutation in COL4A5 gene, X-linked)	1 (3%)
Prune belly syndrome	1 (3%)
Kidney atrophy	1 (3%)
Membranous glomerulopathy	1 (3%)
Interstitial nephritis caused by medication use	1 (3%)
Nephrosclerosis caused by familiar hypercholesterolaemia	1 (3%)
Good pasture syndrome	1 (3%)
Granulomatosis with polyangiitis	1 (3%)

*In 1 case caused by Klinefelter syndrome

Table S3. Baseline characteristics of the donors with pre-donation microscopic hematuria

		Microscopic hematuria	
	Total (n=88)	*Twice before donation (n=68)*	*Once before donation and once after donation (n=20)*
Female sex, n [%]	70 [80]	54 [79]	16 [80]
Caucasian race, n [%]	88 [100]	49 [100]	29 [100]
Age, years	54 (11)	54 (11)	53 (10)
Weight, kg	77 (13)	77 (13)	78 (12)
Height, cm	171 (9)	171 (9)	172 (8)
BMI, kg/m^2	26 (3)	26 (3)	26 (4)
BSA, m^2	1.89 (0.18)	1.89 (0.19)	1.91 (0.16)
SBP, mmHg	125 (11)	126 (11)	122 (12)
DBP, mmHg	75 (9)	75 (9)	73 (8)
Hypertension[∞], n [%]	23 [26]	20 [29]	3 [15]
Use of antihypertensive medication, n [%]	5 [6]	4 [6]	1 [5]
mGFR, ml/min	111 (22)	110 (22)	111 (21)
eGFR, ml/min/1.73m^2	88 (14)	88 (15)	86 (13)
Serum creatinine, µmol/l	72 (11)	72 (12)	73 (9)
Serum glucose, mmol/l	5.3 (0.5)	5.3 (0.5)	5.4 (0.4)
HbA1C, %	5.5 (0.3)	5.5 (0.3)	5.6 (0.3)
Diabetes, n [%]	1 [1]	1 [2]	0 [0]
Serum cholesterol, mmol/l	5.3 (1.0)	5.2 (0.9)	5.6 (1.1)
LDL	3.4 (1.1)	3.3 (1.1)	3.6 (1.5)
HDL	1.7 (0.5)	1.7 (0.5)	1.9 (0.6)
Triglycerides	1.2 (0.8)	1.2 (0.8)	1.3 (0.6)
Serum urea, mmol/l	5.3 (1.2)	5.3 (1.3)	4.9 (1.1)
Serum potassium, mmol/l	3.9 (0.3)	3.9 (0.3)	4.0 (0.3)[a]
Serum sodium, mmol/l	141 (3)	141 (2)	140 (3)
Sodium excretion, mmol/24h	172 (66)	174 (68)	158 (56)
PCR, mg/mmol	9 [0-15]	8 [0-15]	11 [8-14]

[∞]: SBP >140 mmHg and/or DBP >90 mmHg
[a]: P<0.05 vs. "twice before donation" group
Data are presented as mean (standard deviation) for normally distributed variables and as median [first quartile – third quartile] for non-normally distributed variables.
Abbreviations: BMI: body mass index; BSA: body surface area; SBP: systolic blood pressure; DBP: diastolic blood pressure; eGFR: estimated glomerular filtration rate.

Table S4. Long-term follow-up data outcomes

	Moment after donation															
	**3 mo**	**1y**	**2y**	**3y**	**4y**	**5y**	**6y**	**7y**	**8y**	**9y**	**10y**	**11y**	**12y**	**13y**	**14y**	**15y**
**PCR**																
Total population																
Number	363	363	317	279	248	208	144	141	120	94	76	73	39	42	27	10
Median	14	9	9	9	9	8	9	9	9	9	8	9	8	9	9	11
IQR	0-30	6-13	7-13	6-13	6-13	3-14	6-12	7-12	6-13	6-12	6-13	7-12	4-15	7-11	8-17	8-15
Hematuria group																
Number	50	58	45	37	31	18	9	11	8	6	7	6	3	3	4	2
Median	11	10	11	9	11	8	9	8	9	3	8	9	13	8	8	-
IQR	10-28	8-13	6-14	6-13	7-17	4-14	7-13	6-11	2-16	0-14	0-24	7-16	-	-	2-9	-
Non-hematuria group																
Number	313	305	272	242	217	190	135	130	112	88	69	67	36	39	23	8
Median	14	9	9	9	9	8	9	9	9	9	8	8	8	9	10	11
IQR	0-31	6-13	6-13	6-13	6-13	3-14	6-12	7-12	6-12	6-12	6-13	7-12	4-14	7-13	8-17	8-20
**eGFR**																
Total population																
Number	692	597	488	425	360	332	214	180	140	107	103	83	59	53	30	12
Mean (SD)	58 (12)	60 (13)	61 (13)	62 (13)	62 (13)	64 (13)	64 (14)	63 (13)	64 (14)	63 (13)	64 (15)	64 (13)	63 (14)	63 (14)	64 (14)	64 (14)
Hematuria group																
Number	88	77	60	54	44	34	23	14	11	7	8	6	3	7	4	3
Mean (SD)	57±13	59±13	61±15	62±15	63±14	62±13	67±17	66±18	67±16	59±8	66±18	61±10	-	61±17	-	-
Non-hematuria group																
Number	604	520	428	371	316	298	191	166	129	100	95	77	56	46	26	9
Mean (SD)	58±12	60±13	61±13	62±13	62±13	64±13	64±13	63±12	64±14	63±13	63±14	65±13	64±14	63±14	64±14	66±15
**SBP**																
Total population																
Number	687	557	458	390	330	321	199	165	134	104	97	82	57	51	30	14
Mean (SD)	124±13	128±14	130±15	129±14	129±14	128±13	131±16	132±17	131±17	135±16	133±15	135±19	132±16	134±14	134±14	132±15
Hematuria group																
Number	86	77	56	51	44	33	19	15	10	7	6	6	3	6	4	3
Mean (SD)	125±14	130±14	132±15	128±13	131±14	129±12	127±18	132±13	126±18	134±10	132±21	127±15	-	135±17	-	-
Non-hematuria group																
Number	601	480	402	339	286	288	180	150	124	97	91	76	54	45	26	11
Mean (SD)	124±13	128±14	129±14	129±14	129±15	128±13	31±16	132±18	132±17	135±16	133±14	135±19	132±16	134±14	135±15	136±17

Table S5. Long-term follow-up data antihypertensive medication (AH med) use															
	**Moment after donation**														
	**1y**	**2y**	**3y**	**4y**	**5y**	**6y**	**7y**	**8y**	**9y**	**10y**	**11y**	**12y**	**13y**	**14y**	**15y**
**Antihypertensive medication use**															
Total population															
N donors	605	496	433	363	336	218	181	143	108	103	84	59	53	30	14
N (%) AH med use	47 (8)	55 (11)	50 (12)	45 (12)	41 (12)	32 (15)	28 (15)	26 (18)	24 (22)	18 (17)	16 (19)	14 (24)	16 (30)	9 (30)	5 (36)
Hematuria group															
N donors	78	61	55	45	34	24	15	11	7	8	6	2	7	4	3
N (%) AH med use	5 (6)	5 (8)	7 (13)	6 (13)	6 (18)	3 (13)	2 (13)	2 (18)	1 (14)	1 (13)	0 (0)	1 (50)	2 (29)	1 (25)	1 (33)
Non hematuria group															
N donors	527	435	378	318	302	194	166	132	101	95	78	56	46	26	11
N (%) AH med use	42 (8)	50 (11)	43 (11)	39 (12)	35 (12)	29 (15)	26 (16)	24 (18)	23 (23)	17 (18)	16 (21)	13 (23)	14 (30)	8 (31)	4 (36)

Table S6. Linear mixed model analysis for the association between pre-donation hematuria (≥2 RBC per high powerfield or ≥6 RBC per µL) and post-donation PCR, eGFR and SBP over time

	Outcome PCR			Outcome eGFR			Outcome SBP		
	**Estimate**	**95% CI**	**P**	**Estimate**	**95% CI**	**P**	**Estimate**	**95% CI**	**P**
Hematuria[a]	0.13	-0.16 to 0.41	0.38	-1.28	-3.79 to 1.23	0.32	1.80	-0.93 to 4.52	0.20
Time	0.04	0.03 to 0.05	<0.001	0.34	0.24 to 0.45	<0.001	0.95	0.79 to 1.11	<0.001
Hematuria*time	0.02	-0.06 to 0.10	0.50	0.02	-0.51 to 0.54	0.95	0.67	-0.13 to 1.46	0.10

[a]Donors with pre-donation hematuria were defined as 1, donors with no pre-donation hematuria were defined as 0.
Both models were adjusted for pre-donation age, sex, BMI, eGFR, PCR, SBP and antihypertensive medication use.
N total = 701
N hematuria group = 68
N non-hematuria group = 633
Abbreviations: PCR: protein/creatinine-ratio; eGFR: estimated glomerular filtration rate; BMI: body mass index; SBP: systolic blood pressure.

Table S7. Linear mixed model analysis for the association between pre-donation hematuria (≥3 RBC per high powerfield or ≥15 RBC per μL) and post-donation PCR, eGFR and SBP over time

	Outcome PCR			Outcome eGFR			Outcome SBP		
	**Estimate**	**95% CI**	**P**	**Estimate**	**95% CI**	**P**	**Estimate**	**95% CI**	**P**
Hematuria[a]	-0.04	-0.40 to 0.32	0.84	-1.38	-4.33 to 1.57	0.36	1.17	-2.03 to 4.36	0.47
Time	0.04	0.03 to 0.05	<0.001	0.34	0.24 to 0.44	<0.001	0.96	0.80 to 1.12	<0.001
Hematuria*time	0.08	-0.04 to 0.20	0.19	0.15	-0.54 to 0.84	0.68	0.52	-0.49 to 1.54	0.31

[a]Donors with pre-donation hematuria were defined as 1, donors with no pre-donation hematuria were defined as 0.
Both models were adjusted for pre-donation age, sex, BMI, eGFR, PCR, SBP and antihypertensive medication use.
N total = 701
N hematuria group = 46
N non-hematuria group = 655
Abbreviations: PCR: protein/creatinine-ratio; eGFR: estimated glomerular filtration rate; BMI: body mass index; SBP: systolic blood pressure.

Table S8. Linear mixed model analysis for the association between pre-donation hematuria and post-donation PCR, eGFR and SBP over time in a subgroup of donors with hematuria measured twice before donation

	Outcome PCR			Outcome eGFR			Outcome SBP		
	**Estimate**	**95% CI**	**P**	**Estimate**	**95% CI**	**P**	**Estimate**	**95% CI**	**P**
Hematuria[a]	0.22	0.11 to 1.73	0.10	-1.26	-3.71 to 1.18	0.31	2.05	-0.58 to 4.68	0.13
Time	0.04	0.03 to 0.05	<0.001	0.33	-0.23 to -0.44	<0.001	0.95	-0.62 to 0.79	<0.001
Hematuria*time	-0.01	-0.07 to 0.04	0.63	0.22	-0.26 to 0.70	0.38	0.09	-0.62 to 0.79	0.81

[a]Donors with pre-donation hematuria were defined as 1, donors with no pre-donation hematuria were defined as 0.
Both models were adjusted for pre-donation age, sex, BMI, eGFR, PCR, SBP and antihypertensive medication use.
N total = 681
N hematuria group = 68
N non-hematuria group = 613
Abbreviations: PCR: protein/creatinine-ratio; eGFR: estimated glomerular filtration rate; BMI: body mass index; SBP: systolic blood pressure.

Table S9. Baseline characteristics of the living kidney donor population according to presence of risk factors

	Total population (n=701)		High risk subgroup* (n=306)	
	Risk factors*		Microscopic hematuria	
	Present (n=306)	*Absent (n=395)*	*Present (n=41)*	*Absent (n=265)*
Female sex, n [%]	152 [50]	193 [49]	34 [83]	118 [44][b]
Caucasian race, n [%]	306 [100]	395 [100]	41 [100]	265 [100]
Age, years	55 (10)	50 (11)b	56 (10)	54 (10)
Weight, kg	85 (15)	77 (12)[b]	79 (14)	85 (15)[a]
Height, cm	175 (10)	176 (9)	169 (9)	175 (10)[b]
BMI, kg/m^2	28 (4)	25 (3)[b]	28 (3)	28 (4)
BSA, m^2	1.99 (0.21)	1.93 (0.18)[b]	1.90 (0.20)	2.01 (0.21)[a]
SBP, mmHg	131 (15)	123 (10)[b]	126 (14)	132 (15)[a]
DBP, mmHg	78 (9)	74 (8)[b]	74 (10)	78 (9)[a]
Hypertension[∞], n [%]	170 [56]	13 [3]	21 [51]	149 [56]
Use of antihypertensive medication, n [%]	51 [17]	0 [0]	5 [12]	46 [17]
mGFR, ml/min	115 (22)	115 (23)	108 (22)	116 (22)[a]
eGFR, ml/min/1.73m^2	87 (13)	90 (14)[a]	86 (13)	87 (14)
Serum creatinine, µmol/l	141 (2)	78 (13)	72 (12)	79 (14)[a]
Serum glucose, mmol/l	5.4 (0.7)	5.2 (0.5)[b]	5.4 (0.6)	5.4 (0.7)
HbA1C, %	5.5 (0.4)	5.5 (0.3)[a]	5.5 (0.3)	5.5 (0.4)
Diabetes, n [%]	6 [2]	0 [0]	1 [2]	5 [2]
Serum cholesterol, mmol/l	5.4 (1.0)	5.3 (1.1)	5.4 (0.9)	5.4 (1.0)
LDL	3.4 (0.9)	3.6 (0.9)	3.0. (1.0)	3.4 (0.9)
HDL	1.6 (0.6)	1.6 (0.4)	1.8 (0.6)	1.5 (0.6)
Triglycerides	1.5 (0.9)	1.3. (0.9)[a]	1.3 (0.6)	1.5 (0.9)
Serum urea, mmol/l	5.5 (1.3)	5.4 (1.3)	5.3 (1.2)	5.5 (1.4)
Serum potassium, mmol/l	3.9 (0.3)	3.9 (0.3)	3.9 (0.3)	3.9 (0.3)
Serum sodium, mmol/l	141 (2)	141 (3)	141 (2)	141 (2)
Sodium excretion, mmol/24h	191 (72)	199 (73)	155 (59)	197 (73)[a]
PCR, mg/mmol	10 [0-18]	0 [0-9]	13 [7-19]	9 [0-17]

[∞]: SBP >140 mmHg and/or DBP >90 mmHg
[a]: P<0.05 vs "present" group
[b]: P<0.001 vs "present" group
Data are presented as mean (standard deviation) for normally distributed variables and as median [first quartile – third quartile] for non-normally distributed variables.
Donors were classified as high-risk if one or more of the following CKD risk factors were present: SBP>140mmHG and/or use of antihypertensive medication (n=165), eGFR <age-adapted threshold (18) (n=10), PCR>15 mg/mmol (n=100), HbA1c>7% (n=7) or BMI>30 (n=99).
N no risk factors=395; N one risk factor=237; N 2 risk factors=62; N 3 risk factors=7
Abbreviations: BMI: body mass index; BSA: body surface area; SBP: systolic blood pressure; DBP: diastolic blood pressure; eGFR: estimated glomerular filtration rate; PCR: protein/creatinine-ratio.

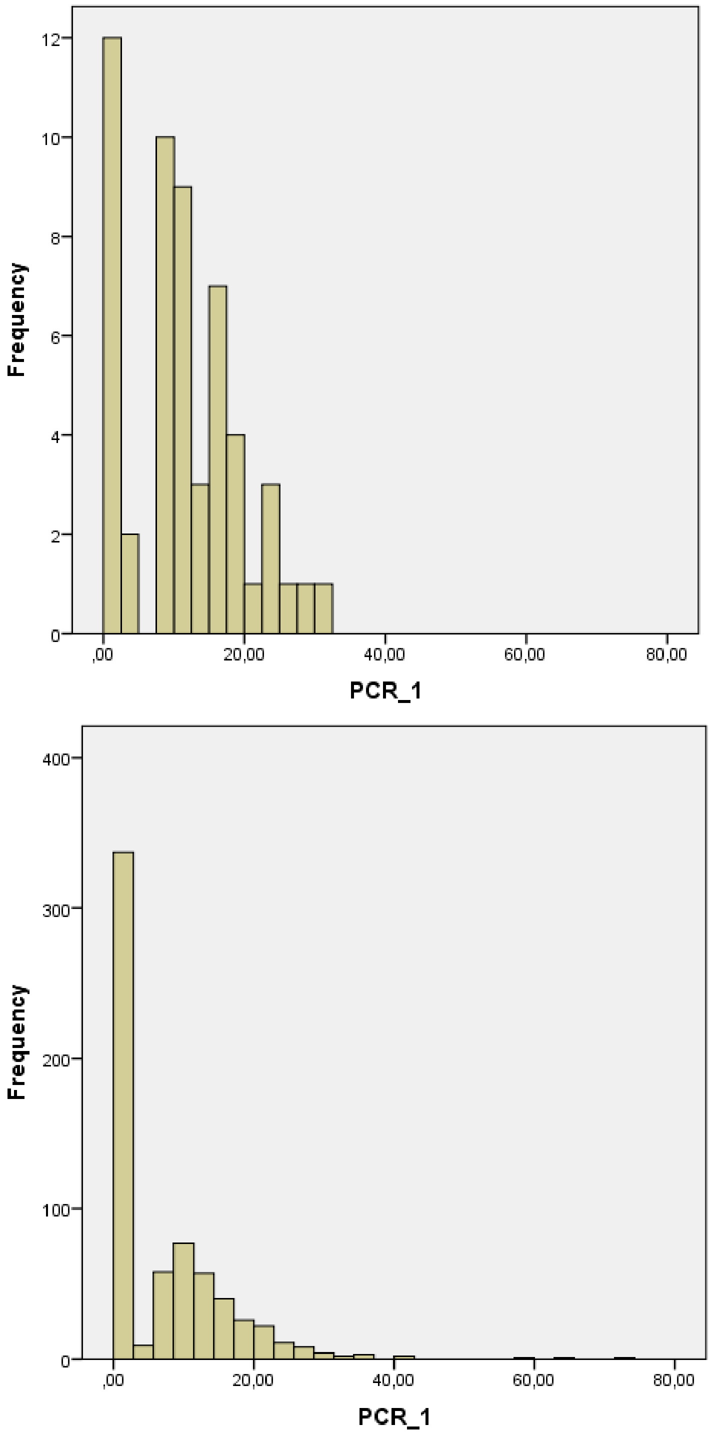

Figure S1. Distribution of pre-donation PCR in the hematuria group and the non-hematuria group. Upper figure: hematuria group, PCR in mg/mmol. Lower figure: non-hematuria group, PCR in mg/mmol.

Supplementary results

Latent class growth analysis

For eGFR and SBP, the best fitting model from the latent class growth analyses was the one with two classes and individual random intercepts and linear slopes (Figures x and y). No clear difference is seen in eGFR decline over time between the two eGFR classes. Also for SBP no worse progression is observed for either class. The best fitting model for PCR was the one with four classes and no individual random effects (Figure z). For the 88 individuals in class 1 PCR increases exponentially after five years after donation, while in the other classes it continues to gradually decrease.

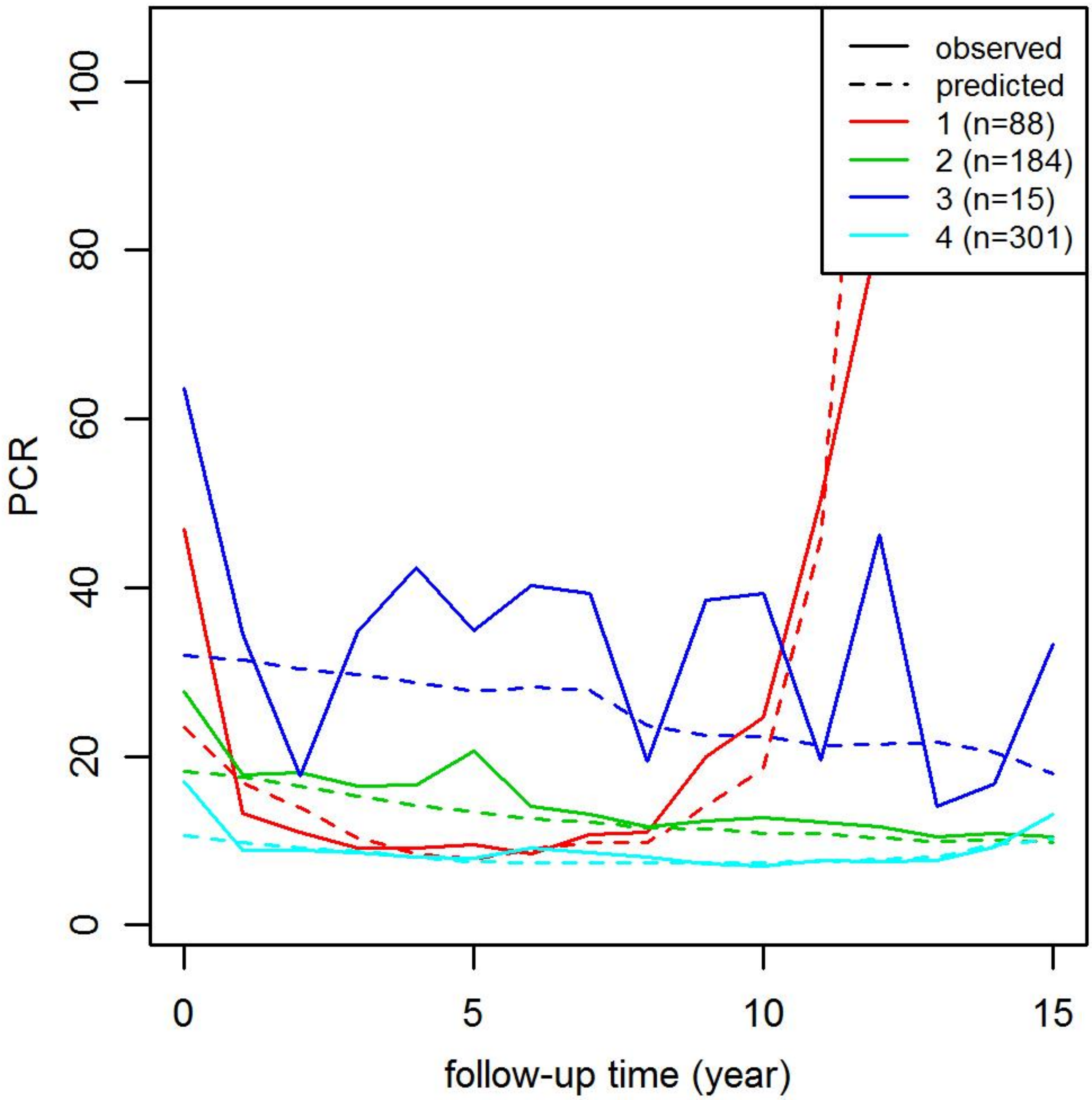

Figure S2. Latent class growth model of post-donation PCR course. The best fitting model was with four classes of post-donation PCR course. We defined group 1 (red) and 3 (dark blue) as “worse” progressors (=1 in logistic regression analysis) and group 2 (green) and 4 (turquoise) as the group with better post-donation outcomes (=0 in logistic regression analysis).

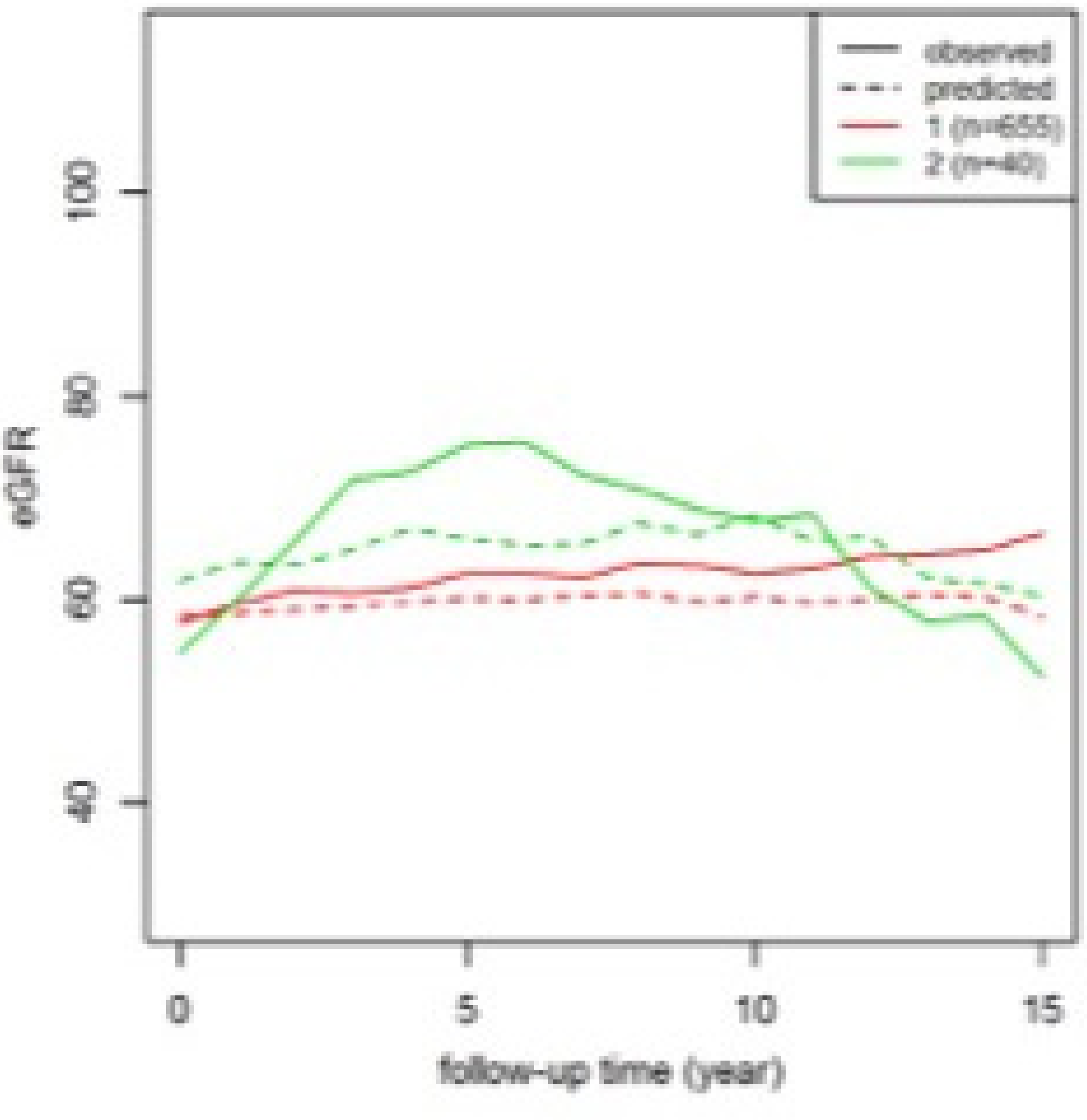

Figure S3. Latent class growth model of post-donation eGFR course. The best fitting model was with two classes of post-donation eGFR course. We defined group 2 (green) as the “worse” progressors (=1 in logistic regression analysis) compared to group 1 (red, =0 in logistic regression analysis).

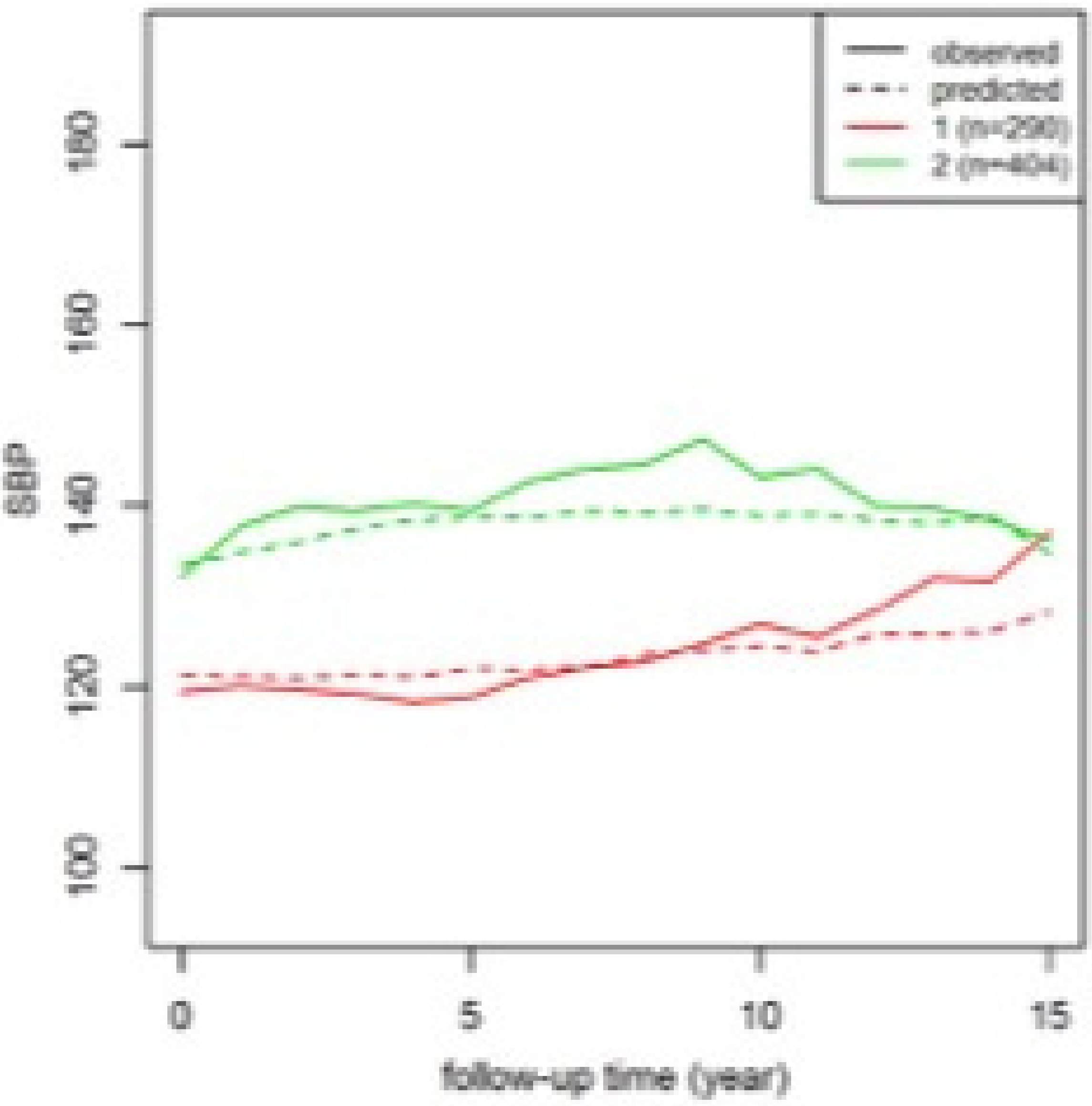

Figure S4. Latent class growth model of post-donation SBP course. The best fitting model was with two classes of post-donation SBP course. Whereas group 2 (green) had a higher post-donation SBP course, the course remained relatively stable. Group 1 (red) showed an increase over time and therefore we defined this group as “worse” progressors (=1 in logistic regression analysis).
